# Characteristics of the polarised off-body channel in indoor environments

**DOI:** 10.1186/s13638-017-0956-6

**Published:** 2017-10-30

**Authors:** Kenan Turbic, Slawomir J. Ambroziak, Luis M. Correia

**Affiliations:** 1 Instituto Superior Técnico, INESC-ID, University of Lisbon, Lisbon, Portugal; 2 Faculty of Electronics, Telecommunications and Informatics, Gdansk University of Technology, Gdansk, Poland

## Abstract

This paper addresses the depolarisation effect in off-body body area networks channels, based on measurements performed at 2.45 GHz in an indoor environment. Seven different scenarios, involving both static and dynamic users, were considered, taking a statistical perspective. The analysis of the cross-polarisation discrimination is performed, as well as the analysis of path loss in co- and cross-polarised channels. Results show a strong dependence of the cross-polarisation discrimination and of channel characteristics on the polarisation and propagation condition, i.e. line-of-sight (LoS), non-LoS or quasi-LoS. Distance, varied between 1 and 6 m in the considered scenarios, is observed to have very little impact on the cross-polarisation discrimination. In the considered dynamic scenario, the channel is characterised by lognormal-distributed shadowing and Nakagami-distributed multipath fading. Parameters of the Nakagami distribution have essentially different values in the co- and cross-polarised channels, showing a trend towards Rice in the former and Rayleigh in the latter. Based on results, a model is proposed for a dynamic off-body channel.

## Introduction

While the depolarisation of an electromagnetic wave transmitted over a wireless channel is a well-known phenomenon, the interest in describing it arose from the fact that orthogonal polarisations can be exploited as additional degrees of freedom in a channel, in order to improve communication quality by means of polarisation diversity [1], or to increase the available data rates by means of polarisation multiplexing [2]. Recently, dual-polarised antennas are being considered for using high data rates in multiple input multiple output (MIMO) systems, when the channel matrix is rank-deficient due to the presence of strong LoS (line-of-sight) [3].

The depolarisation effect in wireless channels yields mismatched polarisations in between the Rx antenna and the impinging E-field, arising from several factors, addressed in what follows. Depolarisation of the LoS component is due to the physical misalignment of the transmitter (Tx) and receiver (Rx) antennas, and also to imperfect antenna cross-polarisation isolation (XPI), where practical antennas inevitably radiate some power in the undesired polarisation other than the one it was designed for (co-polarisation). While this can be avoided in fixed radio links, if antennas’ orientation is carefully chosen, somewhat random antenna rotations in mobile and off-body communications will unavoidably yield variable LoS depolarisation during user’s motion. In addition, interaction with the environment causes additional depolarisation of multipath components (MPCs). According to the geometrical theory of depolarisation [4], the extent of this depolarisation depends on the relative geometry between the antennas and the scattering object, i.e. orientation of the plane of incidence, as well as on the electromagnetic properties of scattering objects, yielding different attenuation and phase changes associated with the orthogonal components of reflected, diffracted, and scattered waves. The channel’s depolarisation characteristics depend on the environment (i.e. its geometry and electromagnetic properties), radiation/polarisation patterns of antennas, propagation conditions (due to the dominance of different depolarisation factors), as well as user’s dynamics.

Several researchers have addressed the depolarisation effect, providing statistical models for the channel depolarisation effects based on measurements, while only few have provided physical models explaining the actual source of depolarisation [4, 5]. An important step in understanding the depolarisation of MPCs was made in [4], where channel coefficients corresponding to orthogonal polarisation components of MPCs at the Rx are obtained from a three-dimensional geometry environment, accounting for Tx and Rx’s relative positions. The model assumes ideally conducting reflection surfaces, therefore, neglecting the depolarisation due to different attenuation of the perpendicular and parallel components. On the other hand, depolarisation due to realistic scattering is modelled in [5, 6]. In [7], the depolarisation effect due to antennas’ mismatch is analysed, where the derivation of the polarisation rotation angle for the LoS component is based on a three-dimensional geometry, for arbitrary orientations of Tx and Rx antennas. The depolarisation of MPCs is modelled by introducing additional factors, depending on cross- and co-polarisation ratios (XPR and CPR, respectively).

The rise of interest in body area networks (BANs) imposed a demand for appropriate channel models, for both on- and off-body scenarios (i.e. communication in between devices along the body, or between the body and an external device), taking BANs’ peculiarities into account, e.g. the proximity of the antennas to the body. With typically low Tx power and on-body antenna rotation during user’s motion, polarisation diversity can provide a valuable means to ensure the required Rx signal quality; optimising system performance requires an accurate polarised channel model. However, the depolarisation effect in BAN channels is somewhat less explored, with most of the available work addressing the on-body case [8–11]. The geometrical theory of depolarisation is applied to obtain a geometry-based on-body channel model [8], where body diffracted, and environment and ground scattered components are taken into account. The same authors also present a modified model [9], which considers the depolarisation due to different Fresnel’s reflection coefficients; one should note that this model considers only static users, since fixed orientations of the Tx and Rx antennas are assumed. The influence of body dynamics is considered in [10], by using animation software to extract motion patterns and apply them to a body phantom in electromagnetic simulations software; as simulations were performed for free space, the authors consider only depolarisation originating from electromagnetic wave interaction with the body, and Tx-Rx antennas’ mismatch due to antenna rotation and tilting during motion.

Very few publications are available on the depolarisation effect in the off-body channel [12–14]. In [12], the authors consider depolarisation due to antenna mismatch, but only basic body rotations are analysed. In [13], an evaluation is performed on the achievable improvement in system performance when dual-polarised antennas are employed at the on-body node, in order to exploit polarisation diversity. Through the analysis of signal-to-noise ratio (SNR) and bit error ratio (BER), it is observed that joint polarisation and spatial diversity can greatly improve system performance. Antenna depolarisation due to the body’s presence is also considered, being observed that Rx field polarisation for linearly polarised Tx antenna becomes elliptical when the antenna is placed on the body. The authors further expand their work in [14], where polarisation and spatial diversity are additionally employed at the off-body side. Neither of the available studies provide an off-body channel model taking the depolarisation effect into account.

While results from the analysis in wireless and on-body channels, and derived models, can give hints on depolarisation effects in off-body communications, its intrinsically different characteristics require dedicated depolarisation studies, providing models that consider the peculiarities of this type of channels. While similarities between mobile and off-body channels clearly exist, the main difference lies in the fact that, in the latter, both Tx and Rx antenna elevations are low and surrounded by scatterers, and that distances are shorter and expected to have a greater impact on depolarisation (according to the geometrical theory of depolarisation [4]). Therefore, the influence of body dynamics is much more significant in the off-body channel than in the traditional mobile one. Similarly, results from on-body channel studies cannot be directly applied to off-body ones, as different propagation mechanisms dominate the two types of body channels. The direction of (linear) polarisation with respect to the body surface is observed to have the greatest influence on the on-body channel, where polarisation normal to the body yields typically much lesser depolarisation and better channel conditions than the tangential one, due to the strong excited surface wave that can propagate around the body as a creeping wave when Tx and Rx are placed on opposite sides of the body [10, 11].

On the other hand, creeping wave propagation mechanisms have almost no influence on off-body propagation, and reflection and scattering in the surrounding environment are the dominating mechanisms. Hence, the polarisation of the propagating signal with respect to the orientation of objects in the propagation environment is important in this case. While scattering in the surrounding environment is present in both on- and off-body channels, different corresponding multipath configurations yield different behaviours of the resulting Rx signal in the two channels. The dependence of the reflection coefficients on the incidence angles [15] is responsible for typically different amplitudes and phases of the reflected MPCs arriving at the Rx in the on- and off-body channels, since these angles are much narrower in the former, as the signal is typically reflected back to the body from nearby objects. Furthermore, different characteristics of the relative motion between Tx and Rx clearly yield a different influence of body dynamics on both types of channels.

The goal of this paper is to investigate the depolarisation effect in off-body channels, based on measurements performed in an indoor propagation environment, considering five scenarios with a static user and different on-body antenna placements, and two others with a dynamic one. The initial results from the measurement campaign were presented in [16]. The work presented in this paper is the extension of the one in [17], with the polarisation of the signal now being taken into account; the statistical analysis of the co- and cross-polarised components (CP and XP, respectively) of the Rx signal is performed, together with the analysis of the cross-polarisation discrimination (XPD). The main contribution of this work is the characterisation of the depolarisation effect and of the individual path loss components in the off-body channel for different polarisations, propagation conditions, and user dynamics. Based on the observed characteristics, a statistical model is proposed.

The rest of the paper is structured as follows: the measurement equipment, procedure and scenarios are described in the following Section 2. Section 3 describes the methodology taken for the analysis, the considered metrics, and the data processing performed to calculate these metrics. The results obtained for XPD are discussed in Section 4, while the results of the statistical analysis are presented in Section 5. A channel model is proposed in Section 6, and the paper is concluded in Section 7.

## Description of scenarios and measurements

### Measurement environment and equipment

**Fig. 1:**
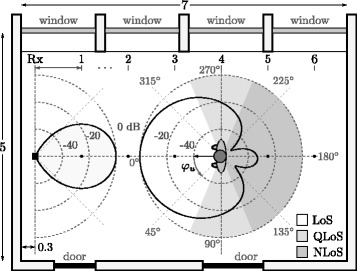
Floor plan of the room, with distances indicated in metres (adopted from [17])

Path loss measurements were conducted at Gdansk University of Technology (GUT), Gdansk, Poland, [18], in a 7 × 5 × 3 m^3^ meeting room with typical objects (tables, chairs, flowers, computers, etc.). The floor plan, shown in Fig. 1, indicates the positions at which measurement samples were collected, the normalised radiation patterns in the azimuth plane of Tx/Rx antennas, and the considered user’s orientations. The following on-body placements (acronyms) have been considered: left side of the head (HE_L), front side of the torso (TO_F), and right-hand wrist (AB_R). These placements were chosen as the representative ones for different antenna motion dynamics, where the chest and head antenna remain fairly steady during user’s motion, while the wrist antenna exhibits significantly more dynamic motion. Moreover, these on-body placements are also representative of popular BAN applications, e.g. smart watches and interactive multimedia glasses. The on-body Tx antenna pattern shown in Fig. 1 corresponds to the TO_F one. One should notice that the orientation of the on-body antenna pattern is fixed with respect to the user’s body, and the antenna rotates together with the user. The relative orientation of the pattern with respect to the body depends on the particular antenna placement, i.e. HE_L, TO_F or AB_R.

Continuous wave (CW) measurements were performed at 2.45 GHz. The Tx section consists of a vector signal generator, R&S SMBV100A [19], a wearable linearly polarised thin micro-strip patch antenna (designed for the operating frequency), and interconnecting cables. The antenna has a 3 dBi gain, and half-power beam-widths of 115° and 140° in the H- and E-planes, respectively. Its small dimensions and flat configuration are suitable for on-body placement.

While the radiation patterns in Fig. 1 were obtained by simulation for free space, placing the antenna on the body primarily introduces an additional attenuation on the back radiation, without significantly changing the front lobe [20]. The connection between signal generator and antenna is done via a 7-m-long RG174 cable [21]. In order to compensate for cable losses, the Tx section was calibrated so that the transmit power at the antenna terminal is 0 dBm (i.e. 3 dBm EIRP).

The Rx section consists of a spectrum analyser, Anritsu MS2724B [22], controlled by a computer that also stores measurement data and performs preliminary calculations. In order to achieve the highest time resolution possible with the available equipment, measurements were performed with the spectrum analyser operating in the variable sampling rate mode, yielding an average sampling period of 150 ms with 40 ms standard deviation. While the measurements’ time resolution is not particularly high, it is considered to be sufficient for the analysis performed in this paper; the environment was free of the moving scatterers, and only static user and low-velocity motion (walk) were considered. The off-body Rx antenna is a horn, LB-OSJ-0760 [23], designed to operate in both polarisations, in the range [0.7, 6] GHz, with a gain of 10 dBi, half-power beam-widths of 58° and 46° in the H- and E-planes, respectively, and a minimum XPI of 36.6 dB. The antenna was put on a 1.4-m-high wooden stand, Fig. 1 (the approximated normalised radiation pattern in the azimuth plane is also being shown). While the used horn is not the typical antenna used in BANs, its characteristics (in the Rx mode) are the appropriate ones for the performed measurements. Switching between vertical (V) and horizontal (H) polarisations is done by using a Tesoel TS121 switch [24]. All RF connections at the Rx are performed with Huber + Suhner Sucoflex cables [25].

### Measurement scenarios

Five scenarios with a static user (S1–S5), one quasi-dynamic (S6), and one true dynamic (S7) ones were considered:

- S1: user standing with Tx antenna at TO_F,
- S2: user standing with Tx antenna at HE_L,
- S3: user standing with Tx antenna at AB_R,
- S4: user sitting in a chair with hands placed on the armrest and with Tx antenna at AB_R,
- S5: user sitting in a chair with hands in reading position and with Tx antenna at AB_R,
- S6: user mimics walking without changing position, with Tx antenna at AB_R,
- S7: user walks across the room, with Tx antenna at TO_F.

For each of these scenarios, measurements were performed for both polarisations, with user’s distance from the off-body antenna varying from 1 to 6 m. Since the analysis of the large-scale effect is of the main interests in static and quasi-dynamic scenarios, i.e. S1–S6, for these scenarios the measurements were performed with the distance being changed with a 1 m step, Fig. 1. At each distance, the user performed a full-body rotation with a 45 ° step (counter-clockwise), and for each body orientation, 50 samples of the Rx power were collected, the corresponding instantaneous path loss values being calculated. In S6, measurements were performed at the same distances as in the static scenarios, while only three user rotations were considered, i.e. 0°, 90°, and 270°. For each body orientation, instantaneous path loss values were recorded for a duration of 45 s. In S7, for each polarisation, measurements were collected for 12 continuous walks across the room over a straight line. In each walk, the user started at 6 m and walked towards the Rx antenna, then turned around after reaching the 1 m point, and walked back to the starting point, Fig. 1.

Measurements were taken with two bodies: B1 (male, 1.76 m height, 88 kg weight) and B2 (female, 1.6 m height, 50 kg weight). Measurements were also performed for the antenna on a dielectric cardboard stand, i.e. without the presence of the body (NB), serving as a reference for the analysis on how the presence of the body impacts on Rx signal polarisation. All scenarios have been investigated for B1, but only scenarios S1 and S4 were taken for B2, and only S1, S4 and S5 for NB. The total number of collected samples is 75,480.

The particularities of each scenario are summarised in Table 1, providing the corresponding Tx antenna height and polarisation, and user orientation angles for which directions of maximum radiations of the Tx and Rx antennas were aligned.

**Table 1:** Characteristics of the investigated scenarios

Scenario	S1	S2	S3	S4	S5	S6	S7
Type	Static					Dynamic	
Antenna placement	TO_F	HE_L	AB_R	AB_R	AB_R	AB_R	TO_F
Tx antenna height [m]	1.3	1.65	0.93	0.79	0.85	0.9 ÷ 1	1.3
Tx antenna polarisation	V			H	45°	V	
φ_max_	0	270	90		90	90	0

In order to facilitate the depolarisation analysis, one has associated polarisations of the Tx antenna to CP and XP channels for each scenario. Obviously, this association depends on the on-body antenna placement and user posture.

Wearable antenna placements in static scenarios S1, S2 and S3 imply that the V polarisation of the Rx antenna yields the CP channel, while the H polarisation corresponds to the XP one. Due to the specific postures, this association is more delicate in S4 and S5; while the on-body antenna is basically H-polarised regardless of user’s orientation, the channel depends on user’s orientation, varying between CP and XP. A CP channel corresponds to antenna polarisations matched to 0° and 180° user orientation angles, while XP is associated with 90° and 270°, the other two being approximately at 45°. Scenario S5 is similar where, depending on the orientation, the channel varies between matched CP and partially depolarised, with antenna polarisations inclined at ± 45° at the extreme.

In scenarios S6 and S7, the geometrical relation between antenna polarisations changes as the user moves. Since movements are somehow periodic and symmetric around the posture of the steady state, the channel polarisation state for the steady state is a logical reference. Since the steady states in S6 and S7 correspond to the static user postures in S3 and S1, respectively, one can consider that the channel is CP for the V polarised Tx antenna in these two dynamic scenarios.

## Analysis methodology

### Extraction of path loss components and data pre-processing

The mean path loss (MPL), shadowing and multipath fading components were extracted from measured instantaneous path loss values, given as [17]:

1 $$ {L}_{\mathrm{PT}}{\left(d,t\right)}_{\left[\mathrm{dB}\right]}=\overline{L_{\mathrm{PT}}{(d)}_{\left[\mathrm{dB}\right]}}+\Delta {L}_{\mathrm{SH}}{(t)}_{\left[\mathrm{dB}\right]}+\varDelta {L}_{\mathrm{MF}}{(t)}_{\left[\mathrm{dB}\right]} $$

where:

- *d*—distance,
- *t*—time,
- $$ \overline{L_{\mathrm{PT}}(d)} $$—MPL component,
- Δ*L*_SH_—shadowing component,
- Δ*L*_MF_—multipath fading component.

The typical log-linear model is used for the MPL component:

2 $$ \overline{L_{\mathrm{PT}}{(d)}_{\left[\mathrm{dB}\right]}}=\overline{L_{\mathrm{PT}}{\left({d}_0\right)}_{\left[\mathrm{dB}\right]}}+10n\kern0.3em {\log}_{10}\left(d/{d}_0\right), $$

where:

- *n*—path loss exponent,
- *d*_0_—reference distance (e.g. 1 m),
- $\overline{L_{\mathrm{PT}}\left({d}_0\right)}$—MPL at the reference distance.

The model’s parameters are estimated by performing a linear regression analysis on the set of path loss values obtained after filtering out the multipath fading. It is important to note that the MPL model fit in this paper differs from the one in [17], since in here the path loss exponent is constrained to a particular value, as discussed at the end of this section.

Due to different measurement procedures, the multipath fading was filtered out differently for the different type of scenarios: in the static and quasi-dynamic cases (i.e. S1–S6), the time average of the instantaneous values was obtained for each distance and user orientation, while for the dynamic one, S7, a moving average filter with a period of 10 wavelengths was applied, as typical for indoor measurements [26]. The averaging distance was calculated based on the average walking speed, varying in between 2.4 and 3 km/h [17], i.e. in between 9 and 13 (typically 11) samples being available for calculating the average, with the sampling rate of the receiver (Section 2.1).

Prior to the statistical analysis, measurement samples were separated into three groups, i.e. LoS, non-LoS (NLoS) and quasi-LoS (QLoS), corresponding to propagation conditions where the direct propagation path between Tx and Rx is unobstructed, fully and partially obstructed by the user’s body, respectively. These groups are intuitively defined with respect to the direction of maximum radiation of the on-body antenna, i.e. always pointing away from the body; the association of direction of departure angles with defined propagation conditions is illustrated in Fig. 1. While the mapping shown in Fig. 1 is independent of antenna placement, association of the propagation conditions with user orientation angles for a particular scenario is easily obtained, given the orientation for which the maximum radiation of the Tx antenna is in the direction of the Rx one (denoted as *φ*_max_ in Table 1). The relative direction of the departure angle *φ*, corresponding to a body orientation angle *φ*_*u*_, is obtained as *φ* = *φ*_*u*_ − *φ*_max_. The absolute mapping is given in Table 2; one should note that, due to the particular orientation of the Tx antenna, all samples in S4 are considered QLoS.

**Table 2:** User orientation angles corresponding to different propagation conditions in scenarios S1–S7

Scenario		S1	S2	S3	S4	S5	S6	S7
*φ*_*u*_ [°]	LoS	0 45 315	225 270 315	45 90 135		45 90 135	90	0
	NLoS	135 180 225	45 90 135	225 270 315		225 270 315	270	180
	QLoS	90 270	0 180	0 180	All	0 180	0	

It is important to point out that the MPL model parameters are estimated in a slightly different way than the traditional one [17], where reasoning for the adopted approach and its advantages over the typical one are discussed in Section 5.6. In this approach, the MPL model, (1), is fitted to the measurements for the reference channel, i.e. LoS case in the CP channel (CP-LoS) of the generalised static scenario, obtained by joining the measurements from scenarios S1 and S2. The obtained path loss exponent for this reference channel is then used as a constraint for the MPL model fit in each considered case, with the MPL value at the reference distance, $\overline{L_{\mathrm{PT}}\left({d}_0\right)}$, being the only estimated parameter. This approach yields a tight fit of the MPL model in the CP-LoS channel, i.e. when the channel is the least affected by propagation phenomena other than path loss, while the goodness of fit (GoF) practically does not change for other propagation conditions (Section 5.6).

### Cross-polarisation discrimination

The most common metric used to characterise depolarisation properties of wireless channels is XPD, calculated as the ratio between the Rx powers received in the CP and XP channels [27], respectively, ${P}_r^{\mathrm{CP}}$ and ${P}_r^{\mathrm{XP}}$, i.e.

3 $$ X={P}_{r\left[W\right]}^{\mathrm{CP}}\kern0.3em /\kern0.3em {P}_{r\left[W\right]}^{\mathrm{XP}}, $$

For a given linear polarisation of the Tx antenna, XPD can be obtained from the path losses observed in the XP and CP channels, e.g. for a V polarised Tx (the superscript in *L*_PT_ indicates the corresponding polarisation):

4 $$ X{\left(d,\varphi \right)}_{\left[\mathrm{dB}\right]}={L}_{\mathrm{PT}}^H{\left(d,\varphi \right)}_{\left[\mathrm{dB}\right]}-{L}_{\mathrm{PT}}^V{\left(d,\varphi \right)}_{\left[\mathrm{dB}\right]}, $$

In the case under analysis, the Tx polarisation depends on the wearable antenna placement and user posture, thus, ensuring Tx-Rx polarisation matching is difficult, namely, in dynamic scenarios. Since, for most of the scenarios, the polarisation of the Tx is practically vertical, the V-polarised Rx antenna is chosen as CP for the calculation of XPD for all scenarios.

The lack of simultaneity in the CP and XP channels constrains the depolarisation analysis only to long-term statistics. Therefore, only the average XPD values are considered in this paper. While some errors are introduced when the XPD is calculated as a ratio of average power levels, the error should be rather small, and the obtained values are a good approximation of the real ones.

In order to investigate the influence of both distance and orientation dependence, XPD is calculated from the composite mean path loss and shadowing component, by averaging over orientation angles, distances, and both, respectively yielding $\overline{X_{\varphi \left[\mathrm{dB}\right]}}$, $\overline{X_{d\left[\mathrm{dB}\right]}}$ and $\overline{X_{\left[\mathrm{dB}\right]}}$. Additionally, the average XPD for the different propagation conditions, i.e. $\overline{X_{\mathrm{LoS}\left[\mathrm{dB}\right]}}$, $\overline{X_{\mathrm{NLoS}\left[\mathrm{dB}\right]}}$ and $\overline{X_{\mathrm{QLoS}\left[\mathrm{dB}\right]}}$, is obtained by averaging over the corresponding subsets of orientation angles associated with each of the propagation conditions (Fig. 1). Finally, one should note that the calculated XPD accounts for both the propagation environment and antenna characteristics.

### Statistical analysis

The statistical distributions considered for path loss components were chosen according to the general knowledge on wireless channels [28, 29], being (the corresponding parameters are indicated as well) [29–31]:

- Rice, with *s*_Rice_ (non-centrality) and σ_Rice_ (scale);
- Nakagami, with *m* (shape) and *Ω* (scale);
- Rayleigh, with σ_Ray_ (scale);
- Lognormal, with *μ*_*L* [dB]_ (log mean) and *σ*_*L* [dB]_ (log standard deviation).

The Rice, Nakagami and Rayleigh distributions are considered for multipath fading, while the lognormal one is for shadowing. The Weibull distribution was also considered for the former, but the fitting results are not included in the paper, as GoF metrics prove that it is not a good fit [17].

Distribution fitting was done with MATLAB’s Statistics and Machine Learning Toolbox [32], i.e. the *fitdist* built-in function, based on maximum-likelihood parameter estimation. In addition, the Akaike information criterion (AIC) [33], *χ* ^2^ and correlation tests [34] were used as GoF metrics as well. While AIC only establishes the relative ordering among the considered distributions, the *χ* ^2^ test gives an absolute measure, indicating if the evidence that samples follow a particular distribution is significant enough. The decision on satisfying a significance is made by comparing the test statistic with a critical value, ${\chi}_{\mathrm{crit}}^2$, determined by the number of bins used for obtaining the empirical PDF from measurements (i.e. 20), number of parameters of the distribution, and required significance, as described in [35] (Sec. 10.4). For a significance level of 5%, the critical value is 27.59 for the Rayleigh distribution, and 28.87 for all the others. Finally, correlation is considered via the coefficient of determination, *R* ^2^, ranging in between 0 and 1, the highest value possible being desirable.

## Analysis of the cross-polarisation discrimination

In order to analyse the depolarisation characteristics of the considered off-body channel, this section presents the XPD values calculated from measurements. The obtained values were analysed with different aspects, with the aim of investigating the influence of different factors on the depolarisation of the transmitted signal. First, the analysis of an average XPD obtained for each scenario is performed to gain an insight into the general polarisation characteristics of the channel, and the influence the wearable antenna placement, user posture and dynamics have on the Rx signal polarisation. The results from this analysis allow the identification of the critical situations for system performance, imposed by the user behaviour, contributing to the selection of the best antenna placements. Second, the comparative analysis of the XPD values obtained when the antenna is attached to the body and when it is placed on the cardboard stand (NB) is performed to get a hint about the contribution of the presence of the body to channel depolarisation, primarily coming from the antenna pattern distortion and the body shadowing. Finally, the influence of the different propagation conditions identified in Section 3.1 are investigated by analysing the XPD values obtained as an average over user orientation angles associated with LoS, QLoS and NLoS (Fig. 1). This analysis reveals the influence of body shadowing on the channel’s polarisation characteristics. Results can serve as a guidance for optimising the design of the distributed spatial diversity systems, intended to ensure the required system performance.

The statistics of XPD calculated from measurements are given in Tables 3 and 4. For each scenario and body (or its absence), Table 3 provides the average XPD for the scenario ($\overline{X}$), and for each propagation condition ($\overline{X_{\mathrm{LoS}}}$, $\overline{X_{\mathrm{NLoS}}}$ and $\overline{X_{\mathrm{QLoS}}}$), while Table 4 gives the corresponding standard deviations (respectively, *σ*_*X*_, ${\sigma}_X^{\mathrm{LoS}}$, ${\sigma}_X^{\mathrm{NLoS}}$ and ${\sigma}_X^{\mathrm{QLoS}}$). It is useful to notice that the obtained average XPD values are considerably lower than the Rx antenna XPI, implying that the used horn antenna is appropriate for the analysis performed in this paper.

**Table 3:** Mean XPD for different propagation conditions and the total average for each scenario

Scenario	Body	$\overline{\boldsymbol{X}}$ [dB]	$\overline{{\boldsymbol{X}}_{\mathbf{LoS}}}$ [dB]	$\overline{{\boldsymbol{X}}_{\mathbf{NLoS}}}$ [dB]	$\overline{{\boldsymbol{X}}_{\mathbf{QLoS}}}$ [dB]
S1	B1	9.73	16.44	6.51	4.50
	B2	9.48	18.43	5.20	2.48
	NB	14.48	20.40	11.95	9.39
S2	B1	5.43	11.71	− 1.25	6.03
S3	B1	4.63	10.09	0.60	2.47
S4	B1	− 2.12	–	–	− 2.12
	B2	−0.79	–	–	− 0.79
	NB	− 0.05	–	–	− 0.05
S5	B1	1.51	3.00	− 0.25	1.90
	NB	− 0.19	− 2.22	0.07	2.46
S6	B1	5.43	7.74	2.20	6.34
	B2	7.04	6.00	3.42	11.70

**Table 4:** Standard deviation of XPD for different propagation conditions and overall for each scenario

Scenario	Body	*σ*_*X*_ [dB]	${\boldsymbol{\sigma}}_{\boldsymbol{X}}^{\mathbf{LoS}}$ [dB]	${\boldsymbol{\sigma}}_{\boldsymbol{X}}^{\mathbf{NLoS}}$ [dB]	${\boldsymbol{\sigma}}_{\boldsymbol{X}}^{\boldsymbol{Q}\mathbf{LoS}}$ [dB]
S1	B1	7.78	2.57	3.94	3.52
	B2	9.96	3.36	5.00	5.37
	NB	8.18	5.84	1.94	5.54
S2	B1	7.61	2.37	3.32	3.00
S3	B1	7.22	1.84	3.73	3.32
S4	B1	9.37	–	–	9.37
	B2	9.60	–	–	9.60
	NB	11.47	–	–	11.47
S5	B1	5.92	1.89	5.00	4.13
	NB	7.41	2.03	4.55	6.49
S6	B1	3.28	2.59	2.80	2.07
	B2	4.07	2.33	2.43	2.29

The average XPD reflects the overall channel’s polarisation characteristics; keeping the focus on the characteristics of each scenario, the measurements for B1 are considered for now. XPD varies from − 2.12 dB in S4 up to 9.73 dB in S1, typically being positive, thus, suggesting that most of the Tx power is contained in the CP channel. Negative values observed in S4 reflect the fact that the particular antenna placement and user posture in this case yield mismatched and effectively orthogonal polarisations of the Tx and Rx antenna polarisations, for most user orientations. Similarly, the posture in S5 is responsible for the values around 0 dB, considering that, in this case, antenna polarisations are typically inclined at 45°. An interesting observation comes from the values obtained for S3 and S6, being characterised by the same wearable antenna placement, the former being a static and the latter a quasi-dynamic scenario. The higher XPD obtained in S6 implies that the channel can actually gain (on average) from user’s dynamics, as the wrist-mounted antenna is periodically brought out of the shadowed region, while it would remain shadowed if the user was static. Clearly, this cannot be considered a rule, and for some cases the opposite can be expected. While not provided in this work, the analysis of the instantaneous XPD would give a more detailed insight into the depolarisation characteristics of the dynamic channel.

By analysing the corresponding standard deviations in Table 4 (*σ*_*X*_), one gets the idea of the variability of XPD over different situations within the same scenario. Very high values, up to 9.36 dB in S4, imply that XPD varies greatly, the reason being because the channel varies from the perfectly CP channel to the orthogonal XP one, as the user rotates. Somehow lower, but still high values obtained for S1–S3 indicate that the depolarisation characteristics of the channel vary considerably, where body shadowing is observed to yield the most significant influence; variations with distance are insignificant. As for the influence of dynamics, interestingly, S6 exhibits lower variations across different orientation angles. Similar to the higher average, this can be attributed to the “softened” NLoS and QLoS cases, i.e. periodical availability of the low-polarised LoS and first-order reflection during the motion cycle, which yields a more stable scenario average.

The body’s influence on XPD, i.e. its presence but also the different body constitutions, can be observed from the available measurements for S1. The difference in between average XPD obtained for B1 and B2, and those for NB, shows the significant influence that the body has on the channel’s polarisation characteristics. The highest XPD value is obtained for the absence of the user (NB), probably being due to the always-present polarisation-matched LoS component and MPCs corresponding to the first-order reflections. As for the different body constitutions, slightly higher XPD values are obtained for B1 compared to B2; however, as the measurements are available only for single representatives of each gender, no conclusions can be drawn from this with confidence.

Considering the average XPD for different propagation conditions, the strong dependence on a particular condition can be observed. Expectedly, LoS propagation yields the highest XPD, with the maximum value of 18.43 dB being observed in S1 (B2); NLoS and QLoS propagation interchangeably yield the lowest XPD values, NLoS slightly more often. This inconsistency on NLoS and QLoS arises from the strong dependence on particular configurations of multipath in the environment, where the constructive interaction of dominant MPCs in the NLoS case can often yield a stronger signal than the direct propagation path in QLoS, considering the low corresponding antenna gain in this case (Fig. 1).

The standard deviations of XPD for different propagation conditions in Table 4 are observed to be lower than the overall for the corresponding scenario (*σ*_*X*_). This confirms that the main variation of XPD within the scenario is between the different propagation conditions. Furthermore, the LoS typically yields a lower standard deviation than NLoS and QLoS, with the latter two having similar values. This is due to the greater sensitivity of XPD to the particular configuration of “visible” scatterers in the absence of the LoS component, since the depolarisation of an MPC is determined by the type, orientation and material of the associated scattering object.

**Fig. 2:**
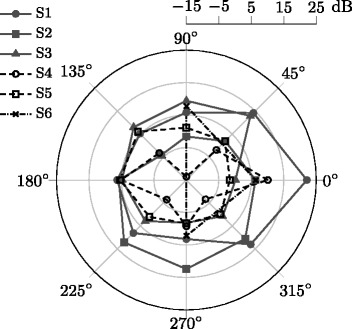
XPD as a function of user orientation for scenarios S1–S6

In order to observe the variations of the XPD within the same propagation condition and wrap up the analysis, it is useful to consider XPD as a function of the user orientation angle, Fig. 2.

It can be noticed in Fig. 2 that the shape of the polygon somehow indicates the user rotation angles associated with different propagation conditions in a particular scenario; the vertices corresponding to the NLoS and QLoS are more rounded and the polygon is slightly pointing into the LoS direction. On the other hand, the polygon area indicates the overall isolation of polarisation components in the considered scenarios. However, this is misleading in the case of S6, as the corresponding polygon is a triangle instead of an octagon, since measurements are available only for three different user orientation angles. Thus, it is clear that the best isolation between CP and XP channels is obtained in S1, for which the shape of the polygon indicates that user rotation angles 0 and ± 45° correspond to LoS.

## Results of the channel analysis

### Initial considerations

Following the analysis of XPD, this section is dedicated to the analysis of the characteristics of the CP and XP channels, considering the statistical properties of the mean path loss and of the shadowing and multipath fading components. Due to their specific characteristics, scenarios S4 and S5 are analysed separately from the others. Regarding the CP channel, the focus is first directed towards the statistics of the (constrained) MPL model fits for each scenario and separately for each propagation condition, where the obtained parameter values, standard deviation and the coefficient of determination are considered. The obtained results give a general insight into the attenuation of the Rx signal dependence on the propagation conditions, the average magnitude of the variations around the mean and how precisely the model describes the attenuation trend with distance. Next, the statistical distributions of the (body) shadowing and multipath fading components are analysed for the dynamic scenario S7, revealing the characteristics of signal variations around the mean.

After the statistical analysis of the channel, the mean path loss at the reference distance and the standard deviation of the signal (around the model-predicted values) are expressed relative to the values corresponding to the CP-LoS channel of the particular scenario. This allows to observe the difference between the CP and XP channels, and the channel characteristics for different propagation conditions.

Finally, the final part of the section is dedicated to the discussion of the particular (constrained) MPL fit chosen in this paper, described in Section 3.1, where the rationale for such a choice is provided. The chosen MPL fit is then compared with the typical and some other possible model fitting choices.

### Co-polarised channel

As described previously, in the MPL fitting approach adopted in this paper, the path loss exponent is constrained to the value corresponding to the reference channel, i.e. the generalised static CP-LoS channel. The obtained value, i.e. *n* = 1.71, being close to that of free space, is therefore common for all scenarios. The statistics of the MPL model parameters obtained with this approach are given in Table 5, showing parameter estimates, coefficient of determination and standard deviations of the signal around the mean.

**Table 5:** Mean path loss for CP/XP channel, body B1 (*n* = 1.71)

		CP			XP		
Scenario	xLoS	$\overline{{\boldsymbol{L}}_{\mathbf{PT}}{\left({\boldsymbol{d}}_{\mathbf{0}}\right)}_{\left[\mathbf{dB}\right]}}$	*R* ^2^	σ_[dB]_	$\overline{{\boldsymbol{L}}_{\mathbf{PT}}{\left({\boldsymbol{d}}_{\mathbf{0}}\right)}_{\left[\mathbf{dB}\right]}}$	*R* ^2^	σ_[dB]_
S1	LoS	31.30	0.75	2.72	47.74	0.72	3.01
	NLoS	48.47	0.12	5.05	54.99	0.04	6.44
	QLoS	42.63	0.02	5.24	47.13	0.39	3.88
S2	LoS	31.67	0.59	3.56	43.38	0.43	4.69
	NLoS	50.83	0.07	6.15	49.57	0.21	4.74
	QLoS	41.66	0.26	4.35	47.69	0.17	5.19
S3	LoS	33.38	0.18	4.70	43.47	0.07	6.02
	NLoS	49.34	0.01	6.80	49.94	0.20	4.35
	QLoS	44.89	0.39	4.83	47.36	0.46	4.68
S6	LoS	32.00	0.54	3.06	39.75	0.80	1.99
	NLoS	49.67	0.00	4.78	51.86	0.77	2.68
	QLoS	41.63	0.69	3.10	47.97	0.94	1.56
S7	LoS	29.80	0.96	0.50	51.03	0.02	3.07
	NLoS	47.67	0.27	2.34	55.20	0.02	2.91

The obtained statistics indicate that the tightest fit is obtained for LoS conditions: the corresponding *R* ^2^ values are the closest to 1, and variance is the lowest. In the QLoS case, where higher standard deviations suggest greater signal variations, the model still follows the general trend with the distance. The loosest fit is obtained for the NLoS case, with the *R* ^2^ values being around zero. By comparing the estimated values of path loss at the reference distance, $\overline{L_{\mathrm{PT}}\left({d}_0\right)}$, it is clearly noticeable that, on average, path loss is the highest for the NLoS case, lower for QLoS case, and the lowest for LoS. Thus, high attenuation and greater variations of the Rx signal should be expected when the body shadows the LoS path.

The statistical analysis of the fading components for the CP channel, i.e. V-polarised Rx antenna, was already performed in [17]; the results show that the lognormal distribution provides a decent fit for the shadowing component in the dynamic scenario S7, while the best fit for multipath fading component was observed to be a Nakagami distribution. It should be emphasised that the Rayleigh distribution always turned out to be a very poor fit, for both users and propagation conditions.

While the adopted approach for the MPL model fit does not have an effect on the multipath fading, it does have one on shadowing. Therefore, the distribution fitting results for the shadowing component in the CP channel slightly differ from those in [17], being presented in Table 6, where columns LogLH, *χ* ^2^ and Corr, respectively, provide values of the log-likelihood, *χ* ^2^ test statistic and correlation coefficient. The mean values around 0 dB imply that the shadowing component is extracted from instantaneous values. Furthermore, the standard deviations are relatively low, suggesting that signal variations due to shadowing are not large in magnitude. However, one has to keep in mind that MPL models were fitted separately for the LoS and NLoS cases, meaning that the attenuation introduced by the full-body shadowing, around 18 dB (Table 5), is contained within the estimated MPL values at the reference distance ($\overline{L_{\mathrm{PT}}\left({d}_0\right)}$), rather than within the shadowing component. Therefore, the variations of the receive signal represented by the shadowing component in this case are primarily due to the movements of the limbs and changes in the way scattering objects MPCs interfere as the user moves along the room, e.g. dominant first-order reflections interchangeably being blocked by the columns (upper wall in Fig. 1) or interacting with the surfaces made of different materials.

**Table 6:** Overview of the lognormal fit for body shadowing component in scenario S7 (${\chi}_{\mathrm{crit}}^2$ is 28.87)

Channel/ Polarisation	Body	Sample size	*μ*_*L*[dB]_	*σ*_*L*[dB]_	LogLH	*χ* ^2^	Corr
CP	B1	772	0.00	1.72	− 379.65	195.22	0.86
	B2	893		2.20	− 658.16	227.18	0.82
XP	B1	736		2.98	− 767.27	34.97	0.99
	B2	865		1.95	− 532.67	15.23	0.99

Shadowing was also analysed for S6, but similarly poor fits are obtained as in [17], thus not being included here. Such results are obtained because the user is not moving through the environment in this quasi-dynamic scenario.

### Cross-polarised channel

The statistics of the MPL model parameters for the XP channel are also shown Table 5, indicating that the model typically fits measurements much more loosely than in the CP channel, suggesting that a greater degree of randomness should be expected. Higher standard deviations, similar to those in the CP-NLoS case, are observed for all propagation conditions, implying that XP channels are characterised by a greater magnitude of Rx signal variations. Moreover, a higher overall average path loss is apparent from $\overline{L_{\mathrm{PT}}\left({d}_0\right)}$ values. Therefore, the lack of a strong signal component in the XP channel is manifested by a lower average received signal power and more severe signal fading than in the CP channel.

The parameters of the lognormal distribution fitted to the shadowing component in the XP channel are also given in Table 6, along with those for the CP one. The values for correlation and *χ* ^2^ indicate that the lognormal distribution is a very good fit. The means are around 0 dB in this case too, and the standard deviation has quite similar values.

**Fig. 3:**
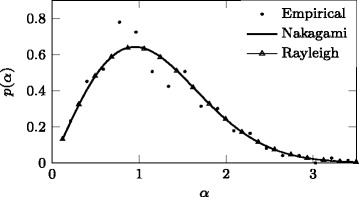
Superimposed Nakagami and Rayleigh PDF fits for multipath component in scenario S7, body B1, NLoS case (cross-polarised channel)

An overview of distribution fitting for the multipath fading component is given in Table 7. It is important to notice, from the *χ* ^2^ test, that all the considered distributions provide a good fit; the 5% significance GoF test is passed in almost all cases, Nakagami distribution being the most common best fit. Since Rayleigh distribution is a special case of the Nakagami one, these results suggest that the Rx signal undergoes somehow Rayleigh fading in the XP channel. This can be visually confirmed in Fig. 3, where the fitted Nakagami and Rayleigh PDFs overlap. Since the Rayleigh distribution implies that multiple-scattered components arrive at the Rx without a dominant component, this fitting was somehow expected, since the XP component antenna only receives depolarised MPCs after reflection and/or diffraction in the propagation environment, while the strong LoS is not detected.

**Table 7:** Overview of distribution fitting for multipath component in XP channel, scenario S7 (${\chi}_{\mathrm{crit}}^2$ is 27.59 for Rayleigh distribution and 28.87 for others)

Scenario	Body	xLoS	Sample	Distribution	Parameters	LogLH	*χ* ^2^	${\boldsymbol{\chi}}_{\mathbf{crit}}^{\mathbf{2}}$	AIC	Correlation
S6	B1	LoS	1800	Rice	*s*_Rice_ = 0.455, σ_Rice_ = 0.921	− 1711.22	216.43	27.59	3426.45	0.82
				Nakagami	*m* = 0.907, Ω = 1.904	− 1705.57	201.53	27.59	3415.15	0.84
				Rayleigh	σ_Ray_ = 0.976	− 1711.35	216.29	28.87	3424.70	0.82
		NLoS		Rice	*s*_Rice_ = 0.049, σ_Rice_ = 0.971	− 1697.73	85.45	27.59	3399.46	0.99
				Nakagami	*m* = 0.917, Ω = 1.889	− 1693.15	75.46	27.59	3390.31	0.99
				Rayleigh	σ_Ray_ = 0.972	− 1697.73	85.50	28.87	3397.45	0.99
		QLoS		Rice	*s*_Rice_ = 0.888, σ_Rice_ = 0.639	− 1383.47	32.19	27.59	2770.95	0.99
				Nakagami	*m* = 1.196, Ω = 1.605	− 1386.61	42.77	27.59	2777.23	0.98
				Rayleigh	σ_Ray_ = 0.896	− 1403.81	73.81	28.87	2809.63	0.96
	B2	LoS		Rice	*s*_Rice_ = 0.778, σ_Rice_ = 0.771	− 1598.39	48.44	27.59	3200.79	0.97
				Nakagami	*m* = 0.9897, Ω = 1.793	− 1603.37	56.50	27.59	3210.75	0.97
				Rayleigh	σ_Ray_ = 0.947	− 1603.44	56.60	28.87	3208.87	0.97
		NLoS		Rice	*s*_Rice_ = 0.103, σ_Rice_ = 0.940	− 1588.98	57.31	27.59	3181.96	0.98
				Nakagami	*m* = 1.002, Ω = 1.779	− 1588.97	57.40	27.59	3181.95	0.98
				Rayleigh	σ_Ray_ = 0.943	−1588.97	57.32	28.87	3179.95	0.98
		QLoS		Rice	*s*_Rice_ = 0.079, σ_Rice_ = 0.942	− 1591.67	49.95	27.59	3187.35	0.98
				Nakagami	*m* = 0.9997, Ω = 1.781	− 1591.67	49.95	27.59	3187.35	0.98
				Rayleigh	σ_Ray_ = 0.944	− 1591.67	49.95	28.87	3185.34	0.98
S7	B1	LoS	346	Rice	*s*_Rice_ = 0.85, σ_Rice_ = 0.67	− 265.85	10.36	27.59	535.73	0.98
				Nakagami	*m* = 1.24, Ω = 1.61	− 263.29	5.86	27.59	530.62	0.99
				Rayleigh	σ_Ray_ = 0.9	− 267.92	15.45	28.87	537.85	0.98
		NLoS	390	Rice	*s*_Rice_ = 0.05, σ_Rice_ = 0.95	− 345.37	9.98	27.59	694.77	0.97
				Nakagami	*m* = 1.01, Ω = 1.79	− 345.36	10.00	27.59	694.76	0.97
				Rayleigh	σ_Ray_ = 0.95	− 345.37	9.97	28.87	692.75	0.97
	B2	LoS	423	Rice	*s*_Rice_ = 0.95, σ_Rice_ = 0.52	− 269.09	43.52	27.59	542.20	0.93
				Nakagami	*m* = 1.53, Ω = 1.45	− 263.42	30.09	27.59	530.88	0.95
				Rayleigh	σ_Ray_ = 0.85	− 284.19	66.58	28.87	570.39	0.88
		NLoS	442	Rice	*s*_Rice_ = 0.91, σ_Rice_ = 0.58	− 306.73	8.60	27.59	617.48	0.98
				Nakagami	*m* = 1.35, Ω = 1.49	− 303.68	6.96	27.59	611.39	0.98
				Rayleigh	σ_Ray_ = 0.86	− 315.20	29.42	28.87	632.41	0.95

Considering the parameters obtained for Nakagami distribution in CP [17] and XP channels (Table 7), multipath fading should be expected to impose greater variability of the Rx signal and deeper fades in the XP channel.

This variability is implied by lower values of the shape parameter *m* and higher values of the scale parameter Ω in the XP case compared to the CP one, considering that *m* is inversely proportional to fading depth and Ω represents the average fading power. This observation is further supported by values of the scale parameter obtained for other distributions, i.e. also corresponding to the fading power, where higher values for parameters σ_Rice_ and σ_Ray_ are always obtained for the XP channel rather than the CP one.

### Scenarios S4 and S5

As discussed in Section 3.2, measurements performed with V- and H-polarised Rx antennas cannot be straightforwardly correlated with CP and XP in scenarios S4 and S5. Specific postures of the user yield effectively a horizontal antenna plane in scenario S4 and 45° inclination of the antenna’s vertical axis in scenario S5, where the actual polarisation of the antenna changes with user’s orientation. In scenario S4, Tx and Rx antennas are co-polarised for user orientation angles (*φ*_*u*_) 0° and 180°, cross-polarised (orthogonal) for angles 90° and 270°, and in between the two for the other orientation angles. In scenario S5, the polarisations of the two antennas vary from matched (co-polarised) to inclined at 45°. Table 8 provides the MPL statistics for the V and H polarisations of the Rx antenna in these two scenarios.

**Table 8:** Mean path loss parameters for V- and H-polarised Rx in scenarios S4 and S5, body B1 (*n* = 1.71)

		V-polarised Rx			H-polarised Rx		
Scenario	xLoS	$\overline{{\boldsymbol{L}}_{\mathbf{PT}}{\left({\boldsymbol{d}}_{\mathbf{0}}\right)}_{\left[\mathbf{dB}\right]}}$	*R* ^2^	σ_[dB]_	$\overline{{\boldsymbol{L}}_{\mathbf{PT}}{\left({\boldsymbol{d}}_{\mathbf{0}}\right)}_{\left[\mathbf{dB}\right]}}$	*R* ^2^	σ_[dB]_
S4	QLoS	45.70	0.30	6.71	43.58	0.31	6.40
S5	LoS	33.81	0.40	3.70	36.82	0.44	3.67
	NLoS	51.14	0.00	8.11	50.88	0.23	5.35
	QLoS	43.58	0.20	4.23	45.48	0.47	5.23

The values obtained for S4 imply that the Rx signal is stronger and variations are smaller for the H-polarised case, which agrees with the fact that in S4 the on-body antenna is in the horizontal plane for all user orientations and its polarisation is mismatched and effectively orthogonal for most of user’s orientation angles. The *R* ^2^ and standard deviations values in S5 are similar for the V- and H-polarised situation in the LoS case, as the Tx antenna inclination of 45° yields practically equal powers in both polarisations. An interesting observation is that *R* ^2^ is the highest for the QLoS case, which should not come as a surprise; the particular on-body antenna placement and body posture yield matched antenna polarisations for user orientations classified as QLoS in S5.

### Channel characteristics relative to the reference

**Fig. 4:**
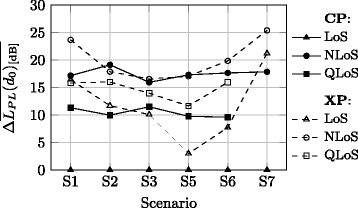
Relative path loss at reference distance

In order to get an idea on how XP antennas and different propagation conditions influence the Rx signal, it is useful to analyse the relative path loss and standard deviation with respect to the CP-LoS case of the corresponding scenario. Figure 4 shows the difference between path loss at the reference distance obtained for a particular polarisation and an xLoS case, and that corresponding to the CP-LoS channel. A clear trend can be observed: for both CP and XP channels, the LoS case yields the lowest path loss, while the highest ones are observed for NLoS. For the CP channel, for LoS approximately 10 and 17 dB higher path loss values are observed compared to the QLoS and NLoS cases, respectively. While the difference is not constant over different scenarios for the XP channel, the relative order is preserved. However, compared to the reference (CP-LoS), these values are typically higher by 10 dB or more; the exception occurs for S5 and S6, which are specific regarding the LoS component mismatch.

**Fig. 5:**
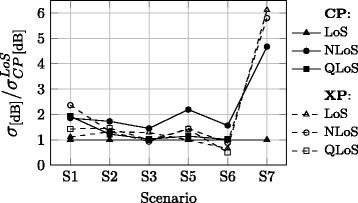
Normalised standard deviation of the signal

Figure 5 shows the ratio of the standard deviation obtained for particular polarisations and xLoS. For static scenarios, the standard deviation almost never increases more than the double of the reference one, while significantly greater differences are observed in the dynamic scenario S7. This is expected, since variability of the signal in static scenarios comes merely from dynamics of objects in the propagation environment and elliptically polarised MPCs associated with reflections from lossy materials, whereas in the dynamic scenario the on-body antenna is moving, rotating and tilting as the user moves.

### Rationale for the adopted approach

**Fig. 6:**
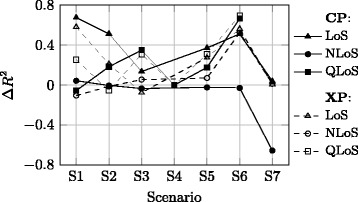
Relative *R* ^2^ values compared to the typical approach

**Fig. 7:**
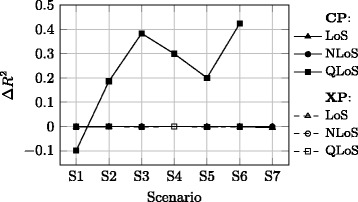
Relative *R* ^2^ values compared to the approach where xLoS are considered separately and no constraint is imposed on *n*

**Fig. 8:**
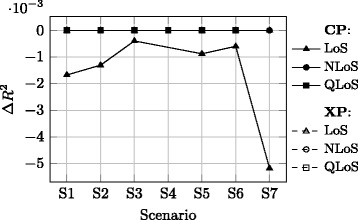
Relative *R* ^2^ values compared to the approach where xLoS are considered separately and *n* is constrained to the value obtained for LoS

Concerning the advantages and disadvantages imposed by adopted approach for the MPL component, Figs. 6, 7 and 8 show the difference Δ*R* ^2^ between *R* ^2^ obtained with this approach and the one when the MPL model is fitted in one of the following ways:

- xLoS cases are taken jointly, and no constraint is imposed on *n*, i.e. the typical approach [17], Fig. 6,
- xLoS cases are considered separately, and no constraint is imposed on *n*, Fig. 7,
- xLoS cases are considered separately, while *n* is constrained to the value obtained for the LoS case in the CP channel for each scenario, Fig. 8.

The positive values in Fig. 6 suggest the chosen approach yields better results than the traditional one in almost all cases, with the most significant improvement observed in the LoS case. This result is the main argument in favour of the chosen approach; MPL model parameters should be estimated for the case when correlation between the path loss and distance is the highest, whereas the variability due to multipath and shadowing effects is imposed by parameters of the corresponding statistical distributions. Notice that the negative values in the NLoS case reflect the fact that the correlation of the Rx signal with distance is reduced when the corresponding samples are considered individually, i.e. without the LoS ones; this is not surprising, since random signal variations due to multipath fading are typically much greater in magnitude than the difference between path loss for Tx-Rx distances between 1 and 6 m.

Figure 7 implies that the better fitting model for the QLoS case in the CP channel is obtained with the adopted approach rather than when the MPL model fit is performed with unconstrained *n* and separately for each xLoS. While Δ*R* ^2^ values for other cases are negative, the difference between the two compared models is negligible in the order of 10^−3^ at maximum.

Furthermore, Fig. 8 implies that the adopted approach yields almost identical *R* ^2^ values as when the model is obtained with *n* constrained to the value corresponding to the CP-LoS channel of the particular scenario. While the difference exists and the maximum is expectedly observed for the LoS case, it is practically insignificant. On the other hand, the adopted approach is clearly less dependent on the scenario.

While these observations justify the adopted approach for the MPL model fitting, the implications it has on the distribution fitting results for shadowing component should be also considered. In order to observe the impact of this choice, it is useful to observe the lognormal distribution parameters obtained in the case the MPL model is fitted according to the conventional approach (as adopted in [17]), Table 9. Comparing these values with those in Table 6, it can be noticed that the adopted approach (Table 6) yields somewhat worse GoF metrics for the CP channel, while GoF is effectively the same for the XP one; even a slight improvement in GoF is observed in the B2 case; however, in general, the difference is not significant.

**Table 9:** Overview of the lognormal fit for body shadowing component in scenario S7, for typical MPL fit approach (${\chi}_{\mathrm{crit}}^2$ is 28.87)

Channel/ Polarisation	Body	Sample size	Parameters	LogLH	*χ* ^2^	Corr
CP	B1	772	*μ*_*L*[dB]_ = 0.00 *σ*_*L*[dB]_ = 1.25	− 134.62	114.84	0.95
	B2	893	*μ*_*L*[dB]_ = 0.00 *σ*_*L*[dB]_ = 1.50	− 314.90	122.22	0.95
XP	B1	736	*μ*_*L*[dB]_ = 0.00 *σ*_*L*[dB]_ = 1.78	− 388.01	30.52	0.98
	B2	865	*μ*_*L*[dB]_ = 0.00 *σ*_*L*[dB]_ = 1.66	− 394.54	17.84	0.99

## Channel model

The statistical channel model proposed for off-body communication represents the instantaneous path loss as a log-sum of three components: the MPL given by (2) and the two random variables corresponding to the shadowing and the multipath fading, i.e.

5 $$ {L}_{\mathrm{PT}\left[\mathrm{dB}\right]}=\overline{L{\left({d}_0\right)}_{\left[\mathrm{dB}\right]}}+10n\kern0.3em {\log}_{10}\left(d\kern0.3em /\kern0.3em {d}_0\right)+\mathrm{N}\left({\mu}_{L\left[\mathrm{dB}\right]},{\sigma}_{L\left[\mathrm{dB}\right]}\right)+20\kern0.3em {\log}_{10}\left\{\mathrm{Nkg}\left(m,\varOmega \right)\right\}, $$

where:

- N—normal distribution,
- Nkg—Nakagami distribution.

The path loss exponent of 1.71 can be taken as a suitable choice for the off-body channel (Section 5.6), while MPL at reference distance depends on polarisation and propagation condition (Fig. 4). A reasonable choice for the reference value corresponding to the CP-LoS channel is 32 dB (Table 5), and the CP-QLoS and CP-NLoS is well-represented if respectively 10 and 18 dB higher values are chosen. While variations between the values across scenarios are considerably greater in the XP channel, a 10 dB higher value than the reference seems appropriate for XP-LoS static scenarios, whereas for dynamic scenario one should take a value 20 dB higher than the reference. Notice that the deviation in S5 is due to specific LoS component mismatch.

As observed in Fig. 5, variability of the signal around MPL also depends on the propagation conditions and polarisation. From Table 5, for the CP-LoS channel a standard deviation of around 3.5 dB seems to be a well-suited choice for static scenarios, and somewhat low value of 0.5 dB for the dynamic ones (Table 5). For the CP-QLoS and CP-NLoS cases in static scenarios, one should expect standard deviations around 1.5 and 2 times greater than the reference one, respectively (Fig. 5). In a dynamic scenario, a considerably greater relative signal variability should be expected in the CP-NLoS case, i.e. around five times higher than for CP-LoS. Again, the values are less consistent across different scenarios in the XP channel. However, for XP-LoS, one should expect a standard deviation that is around 1.2 times greater than the reference in the static scenario, and in XP-QLoS and XP-NLoS cases 1.5 and 2 times higher values, respectively. In the dynamic scenario, a relative increase of six times is common for both XP-LoS and XP-NLoS.

Concerning multipath fading in dynamic scenarios, one should use Nakagami distribution as it can represent all cases; a nearly Rice fading in the CP channel and Rayleigh fading in the XP channel is observed. For the CP channel, values of the shape parameter between 1.2 and 1.3 are suitable for the QLoS and NLoS cases, whereas the value for the LoS case can significantly differ between quasi- and true-dynamic scenarios, i.e. between 4.3 for the former and 19 for the latter. For the XP channel, values between 0.9 and 1.2 could be suitable for the shape parameter in either propagation conditions or user dynamics. Similarly, the subtle differences observed between corresponding values of the scale parameter suggest that values 1.6 and 1.9 will in general provide a satisfactory representation of the multipath fading in this channel.

For the shadowing component, the lognormal distribution should be used in both CP and XP channel, with log mean equal to 0 dB and standard deviation between 1.5 and 3.2 dB.

The ranges of parameters allowing the representation of all considered cases are summarised in Table 10; the reference distance of 1 m is considered. The particular choice for parameters depends on the Tx/Rx polarisation and propagation conditions. While specifying the parameters separately for each propagation condition is somewhat impractical for scenarios where a user moves freely across the environment, as obstructions of the direct propagation path will happen continuously and randomly, many typical scenarios can be characterised by a dominant propagation condition. Such examples are systems for the exchange of information between passengers and a car/plane, and for patients in a hospital.

**Table 10:** Model parameters

*n*	$\overline{{\boldsymbol{L}}_{\mathbf{0}\left[\mathbf{dB}\right]}}$	*m*	Ω	*μ*_*L*[dB]_	*σ*_*L*[dB]_
1.71	[32, 50]	[0.9, 19.5]	[1.0, 2.0]	0	[1.2, 3.0]

It is important to point out the model’s limitations and make some remarks regarding its use and adaptation for different scenarios. One should consider that the model is based on measurements performed in a particular indoor environment. While the chosen environment is typical, the model should be used only for indoor environments of similar size, excluding large halls and outdoor environments. The model should not be used for crowded environments neither, especially in the case where LoS is shadowed by a body other than the user’s. Furthermore, it is important to point out that the model is not applicable to the environments and scenarios in which physical obstructions of the LoS occur.

The model can be used for similar scenarios to the ones considered here, or their combination, which includes users in standing and sitting postures, as well as walking ones. Finally, the adoption of the model to a particular scenario is done by choosing appropriate parameter values. This is done by first identifying the similar scenarios from the ones considered in this paper and then choosing the appropriate value for each parameter from Tables 5, 6, 7 and 8.

## Conclusions

The growing interest in employing BANs for various application requires realistic channel models, allowing the design of low-cost, reliable and energy-efficient systems. Due to specific propagation phenomena and somewhat random movements of users, this is not an easy task. In addition to the random shadowing from the body and constantly varying antenna gain, as antennas tilt and rotate during the movement, the depolarisation of the Rx signal is one of the key effects observed to influence the quality of the channel. Depending on the user’s posture, Tx and Rx antennas can be orthogonally polarised, thus, yielding poor channel conditions which significantly reduce system performance. Therefore, in order to provide a good channel models for BANs, it is important to properly understand this phenomenon, considering the peculiarities of these networks.

With the goal of contributing to the general understanding of the depolarisation phenomenon in BANs, this paper provides the analysis of the depolarisation effect in an indoor off-body channel, based on measurements performed at 2.45 GHz. Both static and dynamic users are considered in a set of seven scenarios, providing the analysis of XPD and statistical analysis of path loss components for different propagation conditions classified according to body shadowing conditions, i.e. LoS, NLoS and QLoS, in two orthogonal polarisations.

Results show a strong dependence of XPD on the user’s orientation, where the highest values are obtained for LoS. While the lowest XPD is observed for QLoS or NLoS interchangeably across scenarios, the NLoS case yields the lowest value more commonly. Furthermore, wearable antenna placement and corpulence of the body show an impact on XPD, higher values being obtained in the case where the antenna is deeper in the shadow region.

A statistical analysis of the signal components in the CP and XP channels implies that the higher attenuation and greater variability of the signal is associated with the latter, as indicated by parameter values of the mean path loss model and distributions of fading components. It is observed that both CP and XP channels exhibit distributed lognormal shadowing and Nakagami multipath fading. However, the parameters of the Nakagami distribution have essentially different values for the two polarisations: the distribution approaches the Rice one in the CP channel, while a tendency towards the Rayleigh one is observed in the XP channel.

Based on the results of the statistical analysis, an empirical model for dynamic off-body channel is proposed, consisting of three components: the mean path loss modelled by a typical log-distance function, the shadowing component modelled by the normal distribution and multipath fading by the Nakagami one. The model can be used to analyse the CP/XP off-body channel for LoS, NLoS and QLoS, by appropriately selecting the values for the parameters.

While the presented work provides valuable insights into the general polarisation characteristics of the indoor off-body channel, the measurements in the CP and XP channels, though performed under the same conditions, were not performed simultaneously. Therefore, future work will involve simultaneous measurements in the CP and XP channels, and the analysis of the instantaneous XPD in order to obtain a more detailed insight into the depolarisation characteristics of the dynamic channel, this being crucial for the investigation of the potential improvements, achievable by means of the polarisation diversity. In addition, some other characteristic environments will be considered.

## Abbreviations

AIC: Akaike information criterion
BAN: Body area network
BER: Bit error ratio
CP: Co-polarised
CPR: Co-polarisation ratio
CW: Continuous wave
GoF: Goodness of fit
H: Horizontal polarisation
LoS: Line-of-sight
MIMO: Multiple input multiple output
MPC: Multipath components
MPL: Mean path loss
NLoS: Non-LoS
QLoS: Quasi-LoS
Rx: Receiver
SNR: Signal-to-noise ratio
Tx: Transmitter
V: Vertical polarisation
XP: Cross-polarised
XPD: Cross-polarisation discrimination
XPI: Cross-polarisation isolation
XPR: Cross-polarisation ratio
